# Gender-Tailored Heart Team Decision Making Equalizes Outcomes for Female Patients after Aortic Valve Replacement through Right Anterior Small Thoracotomy (RAST)

**DOI:** 10.3390/jcdd11100329

**Published:** 2024-10-16

**Authors:** Isabel Lavanchy, Laina Passos, Thierry Aymard, Jürg Grünenfelder, Maximilian Y. Emmert, Roberto Corti, Oliver Gaemperli, Patric Biaggi, Diana Reser

**Affiliations:** 1 Department of Cardiac Surgery and Cardiology, Heart Clinic, Hirslanden Hospital, Witellikerstrasse 40, 8032 Zurich, Switzerland; thierry.aymard@hirslanden.ch (T.A.); juerg.gruenenfelder@hirslanden.ch (J.G.); roberto.corti@hirslanden.ch (R.C.); oliver.geamperli@hirslanden.ch (O.G.); patric.biaggi@hirslanden.ch (P.B.); 2 Department of Cardiac and Vascular Surgery, University Hospital Bern, Freiburgstrasse 20, 3010 Bern, Switzerland; laina.bramazan@hotmail.com; 3 Deutsches Herzzentrum der Charite (DHZC), Department of Cardiothoracic and Vascular Surgery, 13353 Berlin, Germany; maximilian.emmert@access.uzh.ch; 4 Charité-Universitätsmedizin Berlin, 13353 Berlin, Germany

**Keywords:** minimally invasive aortic valve replacement (MIAVR), right anterior small thoracotomy (RAST), minimally invasive aortic valve surgery, aortic valve replacement, gender-related outcomes, aortic stenosis

## Abstract

Background: Little is known about gender-dependent outcomes after aortic valve replacement (AVR) through right anterior thoracotomy (RAST). The aim of our study was to analyze the mid-term outcomes of our cohort. Methods: This study is a retrospective analysis of 338 patients (2013–2022). Subgroup analysis included a gender-dependent comparison of age groups ≤60 and >60 years. Results: Women were older (69.27 ± 7.98 vs. 64.15 ± 11.47, *p* < 0.001) with higher Euroscore II (1.25 ± 0.73 vs. 0.94 ± 0.45, *p* < 0.001). Bypass and cross-clamp time were shorter (109.36 ± 30.8 vs. 117.65 ± 33.1 minutes, *p* = 0.01; 68.26 ± 21.5 vs. 74.36 ± 23.3 minutes, *p* = 0.01), while ICU, hospital stay and atrial fibrillation were higher (2.48 ± 8.2 vs. 1.35 ± 1.4 days, *p* = 0.005; 11 ± 7.8 vs. 9.48 ± 2.3 days, *p* = 0.002; 6.7% vs. 4.4%, *p* = 0.024). Mortality was 0.9%, while stroke was 0.6%. Age subgroup analysis showed that women were older (*p* = 0.025) with longer ICU and hospital stays (*p* < 0.001, *p* = 0.007). On mid-term follow-up (4.52 ± 2.67 years) of 315 patients (94.3%), there was no significant difference in survival, MACCE and re-intervention comparing gender and age groups. Conclusions: Despite older age, higher Euroscore II, longer ICU and hospital stay in women, mortality, MACCE and reoperation were low and comparable in gender and age groups. We believe that our patient-tailored heart team decision making combined with RAST translates into gender-tailored medicine, which equalizes the widely reported negative outcomes of female patients after cardiac surgery.

## 1. Introduction

Minimally invasive aortic valve replacement (MIAVR) is widely accepted and performed as a safe surgical approach for the treatment of isolated aortic valve disease [1]. It offers potential advantages over traditional sternotomy including reduced trauma to the chest bones, decreased blood loss, shorter hospital stay, faster recovery, improved cosmesis, and potentially lower perioperative morbidity and mortality despite longer surgical times [2,3,4,5,6,7]. 

Right anterior small thoracotomy (RAST) is a specialized subgroup of MIAVR which is less accepted and performed, although there are studies showing favorable outcomes compared to sternotomy and mini-sternotomy (less surgical trauma, quicker rehabilitation, less pain and better cosmesis) [8,9,10]. Our clinic has specialized in RAST for isolated aortic valve replacement, because this approach (no-touch sternum) appears to be the most favorable remaining surgical competitor to transcatheter aortic valve interventions (TAVRs). 

Over the past decade, the heart team has evolved to an important entity for patient-tailored treatment according to risk profile and guidelines [11]. The resulting personalized approach ensures that every patient is treated with the lowest risk approach and the most optimal, sustainable and durable outcome [12]. 

Female gender is an accepted independent risk factor for mortality after cardiac surgery [13]. There is increasing evidence that female patients have worse early and late outcomes after aortic valve surgery, especially up to 60 years of age: women often present with advanced stage of aortic valve disease because they might experience atypical symptoms leading to misdiagnosis or delayed diagnosis and treatment [14,15,16]. It could also be shown that women below 60 years have a 2.5-fold higher mortality after cardiac surgery [17]. Little is known about gender and age-dependent outcomes of AVR-RAST. Therefore, the aim of our study was to analyze the mid-term outcomes of our single-center RAST-specialized heart-team-decision-based cohort regarding gender and age-related differences. 

## 2. Methods

### 2.1. Patient Population and Study Design

This is a retrospective, single-center study of all consecutive RAST patients treated at our structural heart team center. We excluded the patients operated on in 2023 and later in order to have more than one year of follow-up for all patients. We collected baseline characteristics, procedural details, in-hospital outcomes, and follow-up data about survival, major adverse cardiovascular and cerebral events (MACCE)-free survival, and freedom from reoperation from our institutional electronic database and follow-up records, and we contacted all patients by phone. Ethics committee approval (approval number 2023-00313) was obtained from our local institutional review board, and signed informed consent was waived. Our structural heart team clinic was established in 2013 with the aim to pioneer patient-tailored medicine. Within our dedicated interdisciplinary heart team, we discusse every valve patient with respect to guidelines and risk profiles and decide on the best treatment option with the least interventional risk (medical versus transcatheter therapy versus cardiac surgery) in order to achieve optimal and sustainable lifetime management. Our heart team meets every week and consists of anesthetists, intensivists, cardiologists (interventionalists, imaging specialists), and heart surgeons. According to our national health care law, patients younger than 75 years do not receive reimbursement for transcatheter aortic valve replacement and therefore have to be planned as surgical candidates unless they are found to be at high risk (>4%) by the heart team or they pay for the intervention themselves. Our center is specialized in minimally invasive heart surgery, including RAST. The following are considered contraindications for this approach: the location of the aorta and the aortic valve to the left of the sternum or low behind the xyphoid, pectus excavatum, concomitant coronary disease requiring grafting, suspected adhesions in the right thorax (irradiation, previous surgery), grade III–IV arteriosclerosis of the ascending and/or descending aorta (porcelain aorta), or a small aortic annulus requiring root enlargement. If one or more of these exclusion criteria are found, the patient is operated via mini-sternotomy or sternotomy, according to the surgeon’s preference.

### 2.2. Surgical Technique

**Figure 1:**
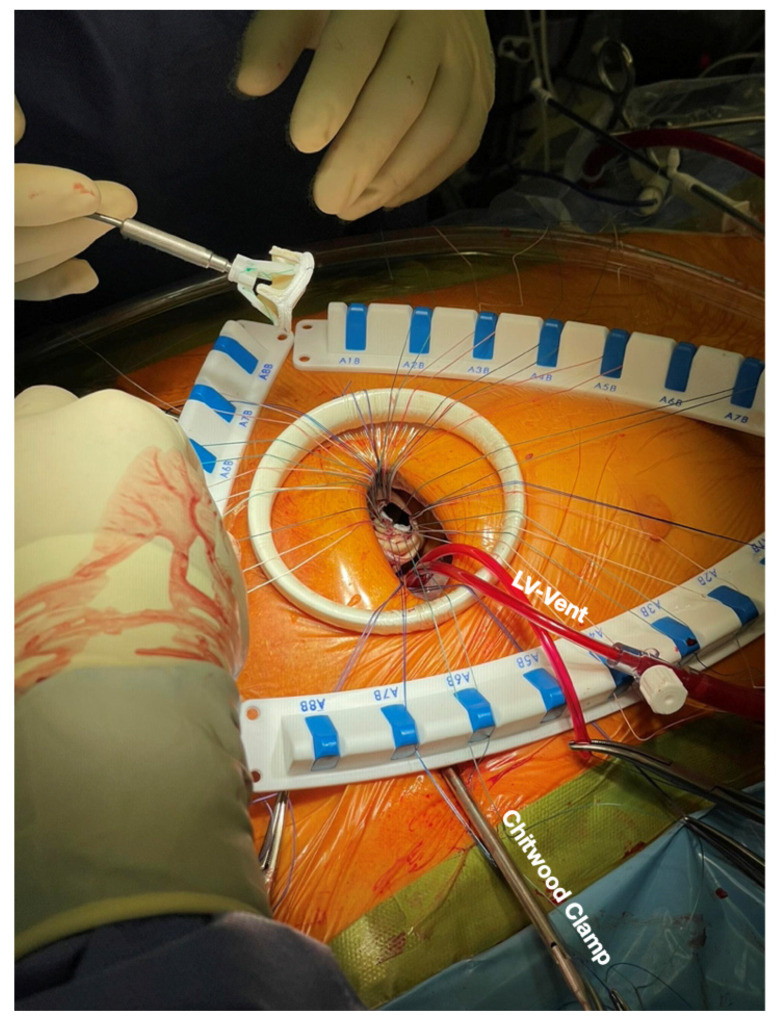
Intraoperative setting of right anterior thoracotomy with left ventricular vent (LV-vent), Chitwood clamp and the interrupted sutures placed in the annulus.

Our surgical technique was published previously in detail [18,19]: right femoral cannulation is performed either percutaneously or surgically through a small cut-down. The thoracotomy is located parasternally in the 2nd intercostal space (see Figure 1). A drain is placed in the 4th intercostal space and is used for CO_2_ insufflation. A left ventricular vent is placed into the right upper pulmonary vein for venting and de-airing. Aortic cross-clamping is performed transthoracically with a Chitwood clamp in the 2nd intercostal space. Cardioplegia is given through an aortic root cannula (Custodiol, Brettschneider). Aortic valve replacement (biological or mechanical) is performed in standard fashion using interrupted braided Teflon felt sutures and the Cor-Knot Device. All patients receive an echocardiogram by our specialized interventional imaging cardiologists in the theater after de-clamping (transesophageal) and before discharge (transthoracic). Biological prosthesis patients take lifelong antiplatelet therapy, and those with mechanical valves are treated with warfarin. For follow-up, all patients are evaluated clinically and echocardiographically by their referring cardiologists 3 and 12 months after surgery and annually thereafter.

### 2.3. Statistical Analysis

All statistical analyses were performed using R version 4.2.1 (The R Foundation for Statistical Computing, Vienna, Austria). Categorical variables are presented as frequencies with percentages and compared between groups using Fisher’s exact test. Continuous variables are presented as mean ± standard deviation (SD) and compared between groups using the t-test or as medians and IQR using the Mann–Whitney test. Overall survival, freedom from aortic valve re-intervention, and freedom from MACCE are presented as Kaplan–Meier curves and compared between groups using the log-rank test. P-values less than 0.05 are considered statistically significant. Because of the small numbers of events, *p*-values are not applicable, and therefore Kaplan–Meier curves for overall survival, freedom from aortic valve re-intervention, and freedom from MACCE in age groups are presented without *p*-values.

Categorical variables were compared between gender adjusted age groups (≤60 and >60 years) using logistic regression. Continuous variables were compared between gender adjusted age groups using linear regression. Variables with skew distributions were logarithmically transformed for this analysis. If no statistically significant interactions werefound, regression analyses without interactions are presented. 

## 3. Results

### 3.1. Baseline Characteristics

Between 30 June 2013 and 31 December 2022 a total of 338 consecutive elective patients underwent AVR-RAST at our clinic performed by four surgeons. In order to analyze gender- and age-dependent outcomes, we defined four groups: male and female, ≤60 and >60 years. Table 1 shows the baseline characteristics of the entire cohort, consisting of significantly more men (71%). Women were older and had a higher Euroscore II (both *p* < 0.001). After mathematically excluding female gender as a risk factor from Euroscore II, there was no more significant differences between male and female patients (*p* = 0.3). However, more women had aortic valve stenosis than men (*p* < 0.001).

### 3.2. In-Hospital Outcomes

Table 2 shows the intraoperative and in-hospital outcomes of the entire cohort. More men received mechanical valves and larger diameter prostheses (*p* = 0.02 and *p* < 0.001). There were three conversions to sternotomy (0.9%); bypass time and cross-clamp time were significantly lower in female patients (both *p* = 0.014), whereas intensive care unit (ICU) and hospital stay were higher (*p* = 0.005 and 0.002). Women had a higher incidence of atrial fibrillation (*p* = 0.024). During hospitalization, there were three deaths (0.9%), two females and one male: a 73-year-old female died on in-hospital day 78 due to sepsis after intraoperative coronary malperfusion followed by sternotomy, the implantation of a smaller valve, bypass grafting and ECMO. The second patient, a 56-year-old female, died on day 8 due to low output. The third patient, a 66-year-old male, was found dead in his bed in the morning of his discharge on day 7 (cause of death unknown, the relatives did not want an autopsy). There were two permanent strokes (0.6%, one in each gender group, *p* = 1). Eight patients needed a rethoracotomy (2.4%), which were performed through the initial incision and ten needed a pacemaker implant during hospitalization (2.9%, *p* = 0.73).

In order to analyze the outcomes below and above 60 years of age, we divided the cohort into two age groups: ≤60 and >60 years (see Table 3). However, women were again significantly older than men (*p* = 0.025), received smaller prostheses (*p* < 0.001) and had longer ICU and hospital stays (*p* < 0.001 and p = 0.007). Atrial fibrillation was at the brink of significance (*p* = 0.055).

### 3.3. Mid-Term Follow Up

The mean follow-up time was 4.52 ± 2.67 years. Follow up of the 335 survivors (99.1%) was completed in 315 patients (94.3%) and closed 31 December 2022 (up to 9.5 years longest follow-up).

**Figure 2:**
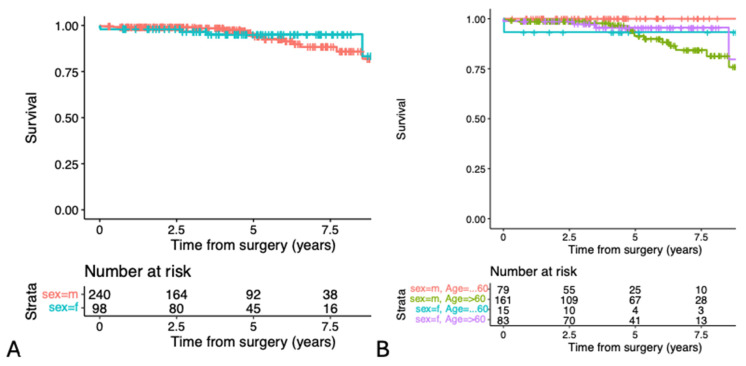
Survival rates by gender (*p* = 0.7) (**A**) and by gender and age groups (≤60 and >60 years) (**B**). Because of the small numbers of events in the age groups, *p*-values are not applicable.

Figure 2A shows the calculated survival rates for male and female patients, which were comparable (*p* = 0.7). Survival at 1 year for women was 98.0% (95% confidence interval (CI) 95.2% to 100%), and for men, it was 99.2% (95% CI 98% to 100%), while the 5-year survival rates were 95.2% (95% CI 90.7 to 100%) and 93.8% (95% CI 89.5 to 98.3%), respectively. There were 19 late deaths (5.7%, 5 women, 14 men due to five cardiac, six non-cardiac and eight unknown causes). 

Figure 2B shows the survival curves of males and females split by age groups, which were comparable (*p* = 0.7).

**Figure 3:**
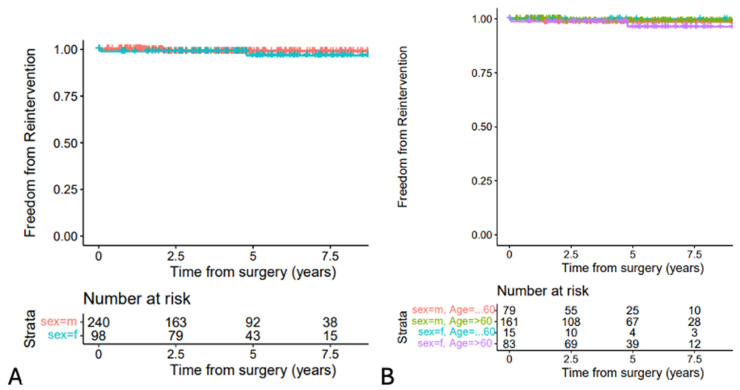
Freedom from aortic valve re-intervention by gender (*p* = 0.4) (**A**) and by gender and age groups (≤60 and >60 years) (**B**) Because of the small numbers of events in the age groups, *p*-values are not applicable.

Figure 3A shows freedom from aortic valve re-intervention for male and female patients, which was comparable (*p* = 0.4). At 1 year, it was 99% (95% CI 96.9 to 100%) for women and 100% for men, and at 5 years, the percentages were 96.8% (95% CI 92.2 to 100%) and 99% (95% CI 97.5 to 100%), respectively. 

Figure 3B shows the calculated aortic valve re-intervention rates of the age groups, which were comparable. Six patients (1.8%) needed a re-intervention for the aortic valve prosthesis: four needed re-do surgery due to endocarditis (day 27, 513, 775 and 1550 after surgery, the last one combined aortic and mitral surgery), and two received a TAVI due to degeneration (day 1445 and 2540). Nineteen patients (5.7%) required a re-intervention for other reasons: one groin revision on day 20, one chronic Type A-dissection on day 1643, one chronic pericarditis on day 116, two mitral valve endocarditis (days 588 and 1550) and 14 pacemaker implants (on days 12, 351, 355, 377, 449, 1096, 1197, 1232, 1259, 1445, 1457, 1603, 1917, 2911). Thirteen of the implanted pacemakers had ICD function (implantable cardioverter–defibrillator). Two patients developed an aortic valve prosthesis thrombosis at days 392 and 1123, which was treated successfully with warfarin.

**Figure 4:**
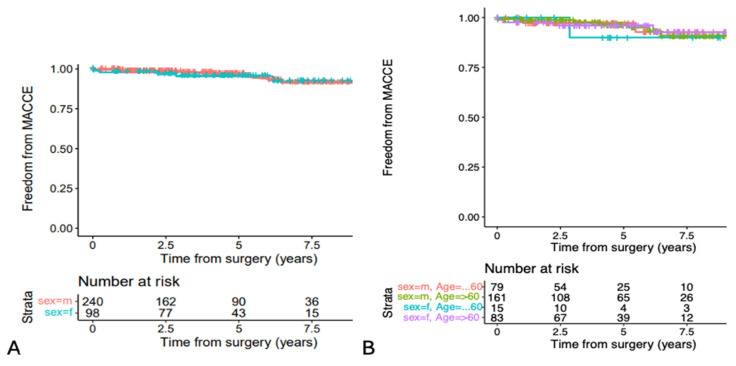
Freedom from MACCE by gender (*p* = 0.8) (**A**) and by gender and age groups (≤60 and >60 years) (**B**) Because of the small numbers of events in the age groups, *p*-values are not applicable.

Figure 4A shows freedom from MACCE of male and female patients, which was also comparable (*p* = 0.8). Freedom from MACCE at 1 year was 97.9% (95% CI 95.1 to 100%) for women and 98.7% (95% CI 97.2 to 100%) for men, and at 5 years, it was 95.4% (95% CI 91 to 99.9%) and 96.8% (95% CI 94.2 to 99.4%), respectively. 

Figure 4B shows the calculated MACCE rates of the age groups, which were comparable. There were three strokes (0.9%) on days 359, 588 (due to mitral valve endocarditis) and 1520. One unstable angina pectoris was reported on day 743 postoperatively (no percutaneous coronary intervention (PCI) required) and in total, there were nine PCIs performed (on days 83, 360, 365, 807, 1120, 1458, 2191, 2196, and 2353).

## 4. Discussion

To our knowledge, this is the first study to investigate gender-dependent outcomes in heart-team selected RAST patients. In our consecutive cohort (N= 338), we could show that mortality, MACCE and need for reoperation were low and comparable between gender and age groups despite older women having a higher Euroscore II as well as longer ICU and hospital stays. We believe that our patient-tailored multidisciplinary heart team decision making combined with RAST translates into gender-tailored medicine, which equalizes the widely reported negative outcomes of female patients after valve surgery despite the relatively low volume of cases of our clinic. 

The perioperative outcomes of our RAST cohort are comparable to or better than those previously published in the literature [5,6,7,8,20,21,22,23,24]: 0.6% stroke, 0.9% conversion to sternotomy, 2.4% re-thoracotomy and 24.2% atrial fibrillation are very similar to the outcomes published by Olds et al. reporting 0.8%, 3.8%, 2.6% and 23.2%, although that study did not use a heart team selection for their RAST patients [22]. Glauber et al. published one of the largest cohorts of RAST patients (N = 593) and also showed comparable results of stroke 1.7%, conversion 2.2%, rethoracotomy 5.1% and atrial fibrillation 25.5% [21]. Our in-hospital mortality is lower (0.89%) then reported in most of the publications ranging between 1.3 and 1.7% [21,23,25]. The mortality reported by Glauber et al. might be the highest but also the most accurate number because of the large number of patients, which translates into statistically relevant results [21]. Among our three deaths, there were two women and one man, which however was statistically not significant, and no further analysis could be performed due to the low event rate.

It is known from the literature that aortic cross-clamp time (ACC) is an independent predictor of morbidity and mortality in cardiac surgery: Swinkels et al. show that a longer ACC is linked to a decrease in late survival with a hazard ratio (HR) of 1.01 for each minute increase in ACC time (95% confidence interval [CI] 1.00–1.02; *p* = 0.012) [24]. On the other hand, cardiopulmonary bypass (CPB) time did not show a significant association with reduced late survival (HR 1.00 per minute increase in CPB time [95% CI 1.00–1.00; *p* = 0.30]) [24]. This finding, however, does not seem to apply to our female cohort: although they had a shorter CPB and ACC (both *p* = 0.014), they had longer ICU and hospital stays (*p* = 0.005 and 0.002) and comparable (not better) short and long-term outcomes. The longer recovery time might be the result of the fact that women were older and sicker at the time of surgery, which however does not seem to translate into worse outcomes. This result may be due to the shorter CPB and cross-clamp times together with the patient-tailored heart team selection. Verheijen et al. were unable to show a correlation between ACC and morbidity and mortality after adjustment for confounding factors in either isolated mitral valve patients (odds ratio 1.04; 95% CI: 0.98–1.11) or concomitant interventions (odds ratio 1.02; 95% CI: 0.97–1.06) [26]. The higher postoperative morbidity and mortality in combined procedures appears to be due to a higher age, more comorbidities, and an extra intervention rather than the duration of ACC [23].

When it comes to mid-term or long-term outcomes of RAST, there are very limited data available in the literature: Glauber et al. reported a survival of 94.8%, MACCE of 13.6% and re-intervention of 1.4% in their follow up study of 31.5 months, which is comparable to our results but in a shorter follow-up period [21]. 

There is increasing evidence that female patients have worse outcomes after cardiac surgery [27]. However, in the past few decades, the interest in performing gender comparing outcome studies in cardiac surgery was very limited to a few research groups. But with the ongoing feminization of the health care system (increasing female med school graduates, female doctors, female surgeons, etc.), this trend seems to change worldwide, and there are great efforts going on to push basic science and clinical trials to include more female subjects in order to analyze the reasons for these negative outcomes. It could be shown that coronary heart disease, the leading cause of global mortality, has historically been under-recognized in women [27]. Women seem to present with different symptoms and therefore have delayed referral or are underdiagnosed (misdiagnosed), have more advanced disease, higher risk due to more comorbidities, are less likely to receive surgery, are older when diagnosed and have higher intraoperative complexity, which automatically translates into higher morbidity and mortality [28]. The awareness of gender medicine was even highlighted by the *New York Times* in January 2024, where an article stated that due to worse outcomes after CABG in women, further research is needed to focus on finding the reasons why [29].

Recent advancements show improved outcomes for women, but further evaluation of interventions like drug-eluting stents and off-pump bypass surgery in women is necessary [27]. Alabbadi et al. analyzed the complications after heart surgery and could show that female patients experiencing complications were older, frailer, and had more comorbidities, whereas men showed a lower mortality rate after complications compared to women even after propensity matching [30]. 

One reason for the negative outcomes in women might be explained by the estrogen depletion: Song et al. could show that perimenopausal women have an increased ischemia–reperfusion injury with a 2.5-fold higher risk adjusted mortality at 40–49 years of age and 2-fold at 50–59 years compared to men [17]. It could be shown that estrogen affects cardiac remodeling in a positive way, which in the future might result in patient-tailored hormone therapy at the time of heart surgery [30]. 

We believe that the heart team decision combined with a specialized team performing minimal invasive valve surgery and catheter-based interventions is crucial for optimal outcomes especially in female patients resulting in patient- and gender-tailored medicine. Our working group showed in a previous study that elderly, small women profit the most from an interventional approach of the mitral valve [31]. We could also show in a propensity matched study that our heart team selects the “best” patients for surgery which might directly result in equal outcomes for female patients with comparable short- and mid-term outcomes [32]. We believe that a heart team approach is easily reproducible in all centers specialized in minimal invasive valve surgery and catheter interventions and should be the standard of care, which ensures the best outcomes. 

However, new approaches always face opposition, such as those described in an English consensus paper stating that the widespread application of RAST should be limited to single-centers because of the elevated technical demands, extended surgery durations, and the challenging learning curve [33]. The heart team implementation has also been discussed controversially due to its potential of conflict of interest of the different specialties due to different budgets, self-interest, loss of autonomy, logistical challenges and resistance to change within professional teams, which may result in biased decision making [34,35,36,37]. 

In summary, our study shows comparable low morbidity and mortality for female and male patients in our RAST cohort despite women being older and sicker. Whether this is the result of the heart team selection process combined with RAST has yet to be further investigated. Future research should focus on identifying variables influencing morbidity and mortality in specific patient subgroups in order to optimize patient and gender-tailored medicine.

## 5. Limitations

The limitations of our study are as follows: we are a RAST-specialized valvular heart team center, and our outcomes might not be reproducible without these preconditions. Our study is observational, retrospective, and single center, which allows for potential biases. We were unable to include control groups, because we do rarely perform median sternotomy for isolated aortic valve surgery and only a few mini-sternotomies. Our RAST patient cohort was low risk, highly selected, included both aortic stenosis and regurgitation patients, and some were lost to follow-up. Furthermore, we did not include our transcatheter aortic valve cohort, because our working group is currently planning a longterm follow-up study of those patients. We did not use any core lab for follow-up echocardiography, which might have resulted in interpretational bias.

## 6. Conclusions

Despite older age, higher Euroscore II, longer ICU and hospital stay in women undergoing AVR-RAST, mortality, MACCE and need for reoperation were low and comparable in both gender and age groups. We believe that our patient-tailored multidisciplinary heart team decision making combined with the minimal invasive approach of RAST translates into gender-tailored medicine, which equalizes the widely reported negative outcomes of female patients after cardiac surgery. However, further studies will be needed to confirm these findings.

## Figures and Tables

**Table 1:** Baseline characteristics of the entire cohort according to gender.

	Female	Male	*p*-Value
Number of patients Age (years)	98 (29) 69.27 ± 7.98	240 (71) 64.15 ± 11.47	<0.001 <0.001
Euro Score II Euro Score II (without gender)	1.25 ± 0.73 1 ± 0.6	0.94 ± 0.45 0.94 ± 0.5	<0.001 0.30
Sinus rhythm	95 (96.9)	231 (96.2)	1
Hypertension	44 (44.9)	90 (37.7)	0.22
Diabetes	9 (9.2)	23 (9.6)	1
COPD	0 (0)	7 (2.9)	0.20
Creatinine > 200 µmol/L	4 (4.1)	7 (2.9)	0.74
LVEF < 30%	1 (1.0)	5 (2.1)	0.68
NYHA III, IV	7 (7.1)	19 (7.9)	1
CVD	3 (3.0)	17 (7.1)	0.21
PVD	0 (0)	9 (3.8)	0.06
Aortic valve stenosis	94 (95.9)	190 (79.1)	<0.001

Mean with standard deviation or number of patients with percentage. COPD = chronic obstructive pulmonary disease, LVEF = left ventricular ejection fraction, NYHA = New York Heart Association, CVD = cerebral vascular disease, PVD = peripheral vascular disease.

**Table 2:** In-hospital data of the total cohort according to gender.

	Female (n = 98)	Male (n = 240)	*p*-Value
Prosthesis	98 (29)	240 (71)	1
Mechanical	1 (1)	18 (7.5)	0.02
Biological	97 (99)	222 (92.5)	1
Size (mm)	22.01 ± 1.4	24.47 ± 1.7	< 0.001
Bypass time (min)	109.36 ± 30.8	117.65 ± 33.1	0.01
Cross clamp time (min)	68.26 ± 21.5	74.36 ± 23.3	0.01
Conversion to sternotomy	1 (0.4)	2 (1)	1
Hospital stay (days)	11 ± 7.8	9.48 ± 2.3	0.002
ICU stay (days)	2.48 ± 8.2	1.35 ± 1.4	0.005
Stroke	1 (0.4)	1 (0.4)	0.497
In-hospital mortality	2 (0.3)	1 (0.4)	0.2
Re-thoracotomy	2 (0.3)	6 (0.8)	1
Red blood cell transfusion	5 (0.7)	13 (1.8)	1
Atrial fibrillation	32 (4.4)	49 (6.7)	0.024
Myocardial infarction	2 (0.3)	0	0.083
Groin seroma	0	4 (0.5)	0.327
Pacemaker implant	2 (0.3)	8 (1.1)	0.73

Mean with standard deviation or number of patients with percentage. ICU = intensive care unit stay.

**Table 3:** Descriptive statistics divided by sex and age group. *P*-values were calculated for the model without interactions between age and gender. The interaction between gender and age groups was not significant (*p* = 0.21). The difference between age groups was significant (*p* < 0.001).

Variable	Age Group	Female	Male	*p*-Value Adjusted for Age Groups
Number of patients	≤60 Years	15 (15.3)	79 (33)	-
	>60 Years	83 (84.7)	161 (67)	-
Age (years)	≤60 Years	54.8 ± 5.5	51 ± 8.9	0.025
	>60 Years	71.8 ± 5	70.6 ± 5.5	“
Sinus rhythm	≤60 Years	0	78 (98.7)	0.57
	>60 Years	80 (96.4)	153 (95.1)	“
Hypertension	≤60 Years	6 (40)	19 (24)	0.46
	>60 Years	38 (45.8)	71 (44.1)	“
Diabetes Mellitus	≤60 Years	0	2 (2.5)	0.45
	>60 Years	9 (10.9)	21 (13.1)	“
COPD	≤60 Years	0	1 (1.3)	1
	>60 Years	0	6 (3.7)	“
Creatinine (>200 µmol/L)	≤60 Years	0	0	0.87
	>60 Years	4 (4.8)	7 (4.4)	“
LVEF <30%	≤60 Years	0	3 (3.8)	0.63
	>60 Years	1 (1.2)	2 (1.3)	“
NYHA III, IV	≤60 Years	2 (13.3)	4 (5.1)	0.73
	>60 Years	5 (6)	15 (9.3)	“
CVD	≤60 Years	0	2 (2.5)	0.98
	>60 Years	3 (3.6)	15 (9.3)	“
PVD	≤60 Years	0	1 (1.3)	0.99
	>60 Years	0	8 (5)	“
Euro Score II	≤60 Years	1.04 ± 0.94	0.75 ± 0.36	0.88
	>60 Years	1.29 ± 0.68	1.03 ± 0.46	“
Aortic valve stenosis	≤60 Years	14 (93.3)	51 (65)	0.62
	>60 Years	80 (96.4)	139 (86.3)	“
Biological Prothesis	≤60 Years	14 (93.3)	63 (80)	0.76
	>60 Years	83 (100)	159 (98.8)	“
Mechanical Prothesis	≤60 Years	1 (6.7)	18 (11.2)	0.18
	>60 Years	0	0	“
Prosthesis Size (mm)	≤60 Years	22.1 ± 1.49	24.5 ± 1.91	<0.001
	>60 Years	22.00 ± 1.39	24.4 ± 1.58	“
Bypass Time (min)	≤60 Years	122.9 ± 48.6	125.3 ± 37.1	0.074
	>60 Years	106.9 ± 26	113.9 ± 30.5	“
Cross clamp time (min)	≤60 Years	74.9 ± 27.4	81.8 ± 27.4	0.06
	>60 Years	67.1 ± 20.1	70.7 ± 20.1	“
Conversion to sternotomy	≤60 Years	0	1 (1.3)	0.83
	>60 Years	1 (1.2)	1 (0.6)	“
Hospital stay (days)	≤60 Years	9.6 ± 2.7	9.1 ± 2.1	0.007
	>60 Years	11.3 ± 8.4	9.7 ± 2.4	“
ICU (days)	≤60 Years	2.0 ± 0.3	1.4 ± 0.1	< 0.001
	>60 Years	2.6 ± 0.2	1.3 ± 0.1	“
Stroke	≤60 Years	0	0	0.63
	>60 Years	1 (1.2)	1 (0.6)	“
Bleeding	≤60 Years	0	4 (5.1)	0.81
	>60 Years	3 (3.6)	3 (1.8)	“
Re-thoracotomy	≤60 Years	1 (6.7)	3 (3.8)	0.99
	>60 Years	1 (1.2)	3 (1.8)	“
Atrial fibrillation	≤60 Years	3 (20)	10 (12.7)	0.055
	>60 Years	29 (35)	39 (24.2)	“
Myocardial infarction	≤60 Years	1 (6.7)	0	0.99
	>60 Years	1 (1.2)	0	“
Groin seroma	≤60 Years	0	1 (1.3)	0.99
	>60 Years	0	3 (1.8)	“
Pacemaker implant	≤60 Years	0	3 (1.3)	0.53
	>60 Years	2 (2.4)	5 (3.1)	“

Mean with standard deviation or number of patients with percentage. COPD = chronic obstructive pulmonary disease, LVEF = left ventricular ejection fraction, NYHA = New York Heart Association, CVD = cerebral vascular disease, PVD = peripheral vascular disease, CVD = cerebral vascular disease, ICU = intensive care unit.

## Data Availability

The raw data supporting the conclusions of this article will be made available by the authors on request.
